# Revisiting the development of *Trypanosoma rangeli* in the vertebrate host

**DOI:** 10.1590/0074-02760240138

**Published:** 2024-11-22

**Authors:** Luan Felipe Santos, Flávia de Souza Rocha, Marcelo Gustavo Lorenzo, Alessandra Aparecida Guarneri

**Affiliations:** 1 Fundação Oswaldo Cruz-Fiocruz, Instituto René Rachou, Grupo de Comportamento de Vetores e Interação com Patógenos, Belo Horizonte, MG, Brasil; 2 Fundação Oswaldo Cruz-Fiocruz, Instituto René Rachou, Grupo de Biotecnologia Aplicada ao Estudo de Patógenos, Belo Horizonte, MG, Brasil; 3 Instituto de Investigaciones en Biodiversidad y Biotecnología, Consejo Nacional de Investigaciones Científicas y Técnicas, Buenos Aires, Mar del Plata, Argentina

**Keywords:** Trypanosoma rangeli, mammal, Chagas disease, infection

## Abstract

**BACKGROUND:**

*Trypanosoma rangeli* is a haemoflagellate parasite that infects triatomine bugs and mammals in South and Central America. *Trypanosoma cruzi*, the etiological agent of Chagas disease, has a partially overlapping geographical distribution with *T. rangeli*, that leads to mixed human infections and cross-reactivity in immunodiagnosis. Although *T. rangeli* can be detected long after mammal infection, its multiplicative forms have not yet been described.

**OBJECTIVES:**

To enhance our understanding of *T. rangeli* development in mammals, this study assessed various infection parameters in mice over time.

**METHODS:**

The parasitaemia, body temperature, and weight of Swiss Webster mice were monitored over 120 days after exposing them to the bites of *Rhodnius prolixus* nymphs containing metacyclic trypomastigotes in their salivary glands. On day 132 post-infection, spleens and mesenteric lymph nodes were analysed for *T. rangeli* DNA using polymerase chain reaction (PCR) and quantitative PCR (qPCR).

**FINDINGS:**

Parasites were detectable in mice blood since day 2 post-infection, detection peaking on day 5 and becoming undetectable by day 120. PCR and qPCR detected *T. rangeli* DNA in the spleens and mesenteric lymph nodes of infected mice. Infected mice showed higher body temperatures and a slower weight gain over time compared to controls.

**MAIN CONCLUSIONS:**

The study confirmed that *T. rangeli* establishes a persistent infection in mice, detectable in lymphoid organs long after parasites had disappeared from blood. In addition, infected mice exhibited physiological changes, suggesting potential subclinical effects. These findings highlight the need for further studies on the immune response and potential impacts of *T. rangeli* infection in mammalian hosts.

*Trypanosoma rangeli* is a trypanosomatid haemoflagellate parasite that infects triatomine insects and mammals in the Americas.^1^ Based on genetic variability, three major lineages of *T. rangeli* have been characterised: KP1(-), KP1(+), and Amazonian,^2^ with most strains showing a strong co-evolution with their sympatric vector species.^3^ ^,^ ^4^ ^,^ ^5^ *T. rangeli* has epidemiological and clinical interest because it shares its geographical distribution and hosts with *Trypanosoma cruzi*, the etiological agent of Chagas disease (CD). Mixed infections of *T. cruzi* and *T. rangeli* have been described in different species of triatomines and sylvatic mammals, including rodents and opossums, as well as in humans.^6^ ^,^ ^7^ ^,^ ^8^ Furthermore, both parasite species exhibit approximately 60% antigenic similarity, leading to immunodiagnostic cross-reactivity.^9^ ^,^ ^10^ ^,^ ^11^ ^,^ ^12^ Although infection by *T. rangeli* elicits high antibody levels, it apparently does not cause a human disease.^13^

*Trypanosoma rangeli* induces different levels of pathogenicity in the invertebrate host, this is especially true for *Rhodnius* species, since their salivary glands can be invaded. Indeed, this is the only triatomine genus in which *T. rangeli* is able to produce infective forms.^14^ ^,^ ^15^ The development of *T. rangeli* in triatomines begins after a blood meal from an infected mammal. Blood trypomastigotes differentiate into replicative epimastigotes in the anterior midgut of the bugs and within a few weeks, the entire intestinal tract is colonised.^16^ Eventually, parasites cross the intestinal epithelium and begin multiplying in the haemocoel of bugs. Later, they can invade the salivary glands and transform into metacyclic trypomastigotes. The latter is the form transmitted through the triatomine bug’s bite to the mammal host during a subsequent blood meal.^17^ The development of *T. rangeli* in mammals is still the focus of debate, as little is known about its survival and multiplication in these hosts. *In vitro* studies using different cell lineages failed to demonstrate replicative forms, suggesting that the parasite does not multiply intracellularly.^18^ ^,^ ^19^ ^,^ ^20^ Nonetheless, a recent study revealed the presence of both *T. rangeli* DNA and live parasites in the spleen and mesenteric lymph nodes of infected mice 30 days after they were infected through the bite of triatomines containing metacyclic forms in the salivary glands.^21^ Furthermore, positive xenodiagnosis and haemoculture from chronically infected mammals, as well as human patients, reinforce the idea that *T. rangeli* maintains a developmental cycle in the vertebrate host.^22^ ^,^ ^23^ ^,^ ^24^ In this sense, the objective of this study was to assess different parameters of *T. rangeli* infection in Swiss mice during a four-month infection interval, following exposure to the bites of *Rhodnius prolixus* nymphs containing metacyclic trypomastigotes in the salivary glands.

## MATERIALS AND METHODS

*Organisms* - The *T. rangeli* Choachi (KP1(+)) strain, originally isolated from naturally infected *R. prolixus*,^13^ was utilised in this study. The parasites were cultured through biweekly passages in liver infusion tryptose (LIT) medium supplemented with 15% foetal bovine serum, 100 U/mL penicillin, and 100 μg/mL streptomycin. The infectivity of the strain was maintained by exposing the parasites to triatomines and mice every three months.^25^

The *R. prolixus* nymphs used to infect mice with *T. rangeli* were obtained from a laboratory colony maintained by the Grupo de Comportamento de Vetores e Interação com Patógenos at the Instituto René Rachou. Insects were fed monthly on citrated sheep blood (Ins-tituto de Ciência e Tecnologia em Biomodelos - ICTB - Fiocruz, Rio de Janeiro, Brazil) offered through an artificial feeder at 37ºC.

Swiss Webster mice, approximately 60 days old and weighing around 40 g, were obtained from an in-house animal care facility and served as the vertebrate host model of this study. Mice were intraperitoneally anesthetised with a mixture of Ketamine 150 mg/kg (Cristalia, Brazil) and xylazine 10 mg/kg (Bayer, Brazil).

*Mice infection* - Mice were weighed, anesthetised, and randomly divided into two groups. The first group was exposed to the bites of 10 *R. prolixus* nymphs carrying metacyclic *T. rangeli* in their saliva for approximately one minute (n = 10). The second group served as a control and was bitten by uninfected nymphs for the same duration (n = 10).

*Parameters evaluated* - The parasitaemia of infected mice was assessed by collecting 5 µL of blood from mice tails on days 1-14, 30, 60, 90, and 120 following infection with *T. rangeli*.^26^ Blood was also collected from the uninfected group to standardise animal conditions. Prior to blood collection, each mouse was weighed, and its body temperature measured using a thermo imager (240 x 180 pixels, Testo 871, Titisee-Neustadt, Germany). Temperature readings were taken with the mouse held in a cylindrical plastic container (10 cm high x 8 cm in diameter), maintaining approximately 20 cm between the thermo imager and the animal. The captured images were analysed using Testo IRSoft Software 5.0, which enables the selection of different points/areas on the animal body surface. The warmest temperature point (located in the mouse head), and the mean body temperature (excluding the tail) were used to compare infected and uninfected mice. Blood sample collections and temperature measurements were conducted at the same time each day (2:30 PM). On day 132 post-infection, the body temperature of the mice was measured, and they were then anesthetised and exposed to two third instar *R. prolixus* nymphs. Following this, blood samples (~ 0.8 mL) were collected and transferred to tubes containing 2 mL of LIT. Subsequently, mice were euthanised, and their spleen and mesenteric lymph nodes excised with the aid of autoclaved forceps and scissors. Afterwards, tissues were weighed and transferred to microtubes containing PBS (0.15 M sodium chloride in 0.01 M sodium phosphate, pH 7.4). Samples were stored at -20ºC until the DNA extraction.

The extraction of *T. rangeli* DNA from spleen and mesenteric lymph nodes was performed using the Puregene Core Kit A (QIAGEN Sciences, Maryland, USA), according to product instructions. Tissues were prepared according to Ferreira et al.,^21^ with modifications. The mesenteric lymph nodes and spleens were transferred to tubes containing lysis solution, cut into small fragments and macerated using glass beads (Sigma-Aldrich, Missouri, US) in a Mini Bead-Beater 96 (BioSpec Products Inc., Bartlesville, OK, US). After incubation at 65ºC for 30 min, the samples were incubated with proteinase K at 55ºC for 20 h (Promega Corporation, Madison, USA). DNA concentration was measured with a NanoDrop™ One Microvolume UV-Vis Spectrophotometer (Thermo Scientific, Wilmington, USA) and adjusted to a concentration of 20 ng/µL. DNA was also extracted from culture epimastigotes (1 x 10^7^ par/mL) as described and used as a positive control.

*DNA amplification* - The following pair of primers was used to amplify a 105 bp fragment of the *T. rangeli* annotated KMP-11 gene: KMP84_F: GAAGTTCTTTGCGGACAAGC and KMP188_R: TTGAACTTGTCGGTGTGCTC.^21^ A second band of approximately 600 bp was also amplified in some of the samples and corresponds to two copies of the KMP-11, as shown by Diez et al.^27^ Polymerase chain reaction (PCR) reactions were carried out in a volume of 12.5 µL comprising: 7.65 µL of DNAse/RNAse-free water, 2.5 µL of 5x Gotaq Buffer (Promega) 0.5 µL of each primer (10 mM), 0.25 µL of dNTPs 10 mM (Invitrogen, Massachusetts, US), 0.1 µL of Gotaq Polymerase (Promega) and 1 µL of DNA. The conditions used were 30 s at 94ºC, followed by 40 cycles of 45 s at 94ºC, 45 s at 60ºC, 1 min at 72ºC, and final extension for 4 min at 72ºC. The PCR product was resolved in agarose gel at a concentration of 2%, stained with Gel Red, with a final concentration of 0.5 μg/mL.

For quantitative PCR (qPCR), each reaction was carried out in a final volume of 10 µL containing 1 µL of DNA (20 ng), 0.5 µL of each primer (10 μM), 5 µL of Power SYBR Green PCR Master Mix (Applied Biosystems, Waltham, USA) and 3 µL of DNAse/RNAse-free water. On each plate, controls consisted of three wells of uninfected spleen or lymph node samples, three no template controls, and three wells of *T. rangeli* DNA from cultured parasites. The conditions used were 10 min at 95ºC, followed by 40 cycles of 15 s at 95ºC and 1 min at 60ºC. Samples were analysed in duplicate. An analysis of the dissociation curve (Tm) was done to confirm the specificity of the reaction. Samples were considered positive when amplification occurred at the same melting temperature as the sample containing *T. rangeli* DNA form culture parasites. Reactions were performed in a ViiA 7 Real-Time PCR System (Applied Biosystems, Foster City, California-USA) at the qReal-Time PCR Facility-RPT09D PDTIS/Instituto René Rachou/FIOCRUZ, MG.

*Statistical analysis* - The effects of time on parasitaemia, as well as the effects of time, infection, and their interaction on body temperature and weight were analysed using generalised estimation equations (GEE). Survival was evaluated using the Log-rank test. In all statistical tests performed in this study, the level of significance was set to α ≤ 0.05.

*Ethics* - All experiments involving live animals were performed following FIOCRUZ guidelines on animal experimentation and were approved by the Comissão de Ética no Uso de Animais de Laboratório (CEUA/FIOCRUZ) under approved protocol number LW 03/22. The protocol is from Conselho Nacional de Controle de Experimentação Animal CONCEA/MCT (https://www.gov.br/mcti/pt-br/composicao/conselhos/concea), which is associated with the American Association for Animal Science (AAAS), the Federation of European Laboratory Animal Science Associations (FELASA), the International Council for Animal Science (ICLAS) and the Association for Assessment and Accreditation of Laboratory Animal Care International (AAALAC).

## RESULTS

**Fig. 1:**
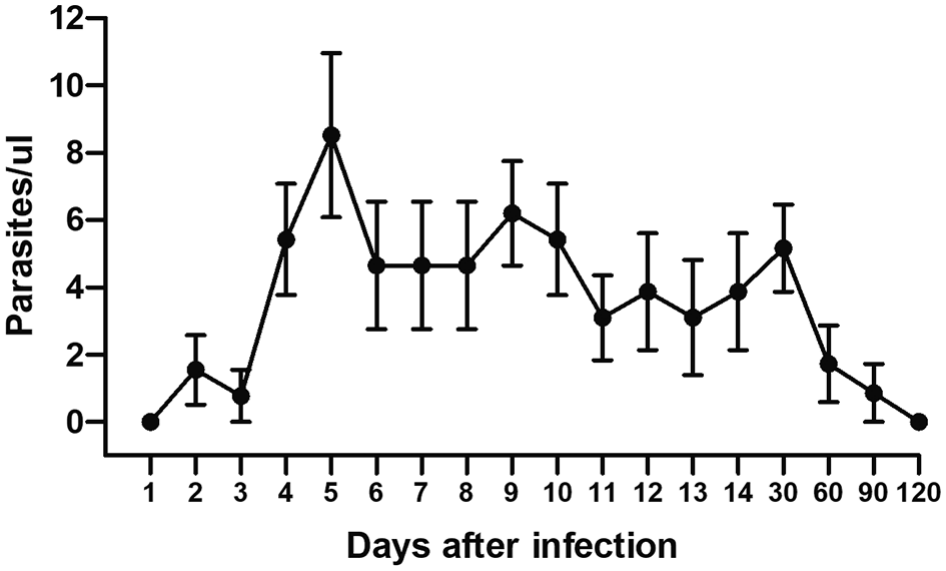
number of circulating *Trypanosoma rangeli* in mice blood over 120 days of infection. Mice were infected through the bite of *Rhodnius prolixus* nymphs containing metacyclic trypomastigotes in their salivary glands. Data are shown as the mean ± s.e. [generalised estimation equations (GEE), p = 0.018; n = 7-10 for each treatment].

**Fig. 2:**
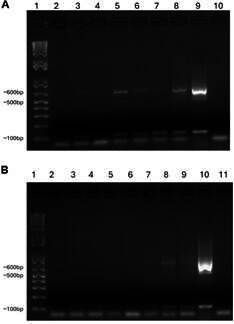
agarose gel electrophoresis (2%) of polymerase chain reaction (PCR) amplicons after amplification of *Trypanosoma rangeli* KMP-11 fragments in (A) mesenteric lymph nodes and (B) spleen of mice infected through the bite of *Rhodnius prolixus* nymphs containing metacyclic trypomastigotes in their salivary glands. (A) Lane 1: 1 kb DNA ladder; Lane 2: uninfected mouse; Lanes 3-8: *T. rangeli*-infected mice; Lane 9: cultured *T. rangeli*; Lane 10: negative control without DNA. (B) Lane 1: 1 kb DNA ladder; Lane 2: uninfected mouse; Lanes 3-9: *T. rangeli*-infected mice; Lane 10: cultured *T. rangeli*; Lane 11: negative control without DNA.

*Time course of parasite presence* - Parasites appeared in blood samples from day 2 post-infection (p.i.), with parasitaemia peaking on day 5 p.i. (8.28 ± 2 par/µL) and decreasing over time until no parasites were found on day 120 p.i. (Fig. 1). According to the GEE model, the mean parasitaemia decreased 0.0236 units each time point (GEE; Wald 5.59, p = 0.018). Both xenodiagnosis and haemoculture from blood collected on day 132 p.i. were negative. *T. rangeli* DNA was detected in both organs of all the seven infected animals using PCR and/or qPCR, except for the spleen of mouse #3, where no parasite DNA was detected by any technique (Fig. 2, Table I). Therefore, according to our results, parasites disappear from the circulation between 90 and 120 days, but organ infection persists.

**TABLE I:** Presence of *Trypanosoma rangeli* DNA in samples of mesenteric lymph nodes and spleen of mice exposed to the bite of *Rhodnius prolixus* nymphs containing trypomastigote forms in their salivary glands

Mouse	Mesenteric lymph nodes		Spleen	
	PCR	qPCR	PCR	qPCR
1	+	+	+	+
2	-	+	+	+
3	+	+	-	-
4	+	+	-	+
5	^*^	^*^	+	+
6	+	+	+	+
7	+	+	+	+

* The mesenteric lymph nodes of mouse 5 were lost during dissection. PCR: polymerase chain reaction; qPCR: quantitative PCR.

**Fig. 3:**
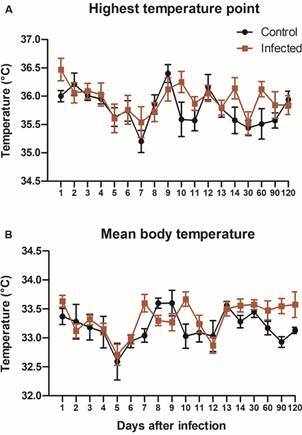
*Trypanosoma rangeli* infection increases the body temperature of mice. Mice were infected through the bite of *Rhodnius prolixus* nymphs containing metacyclic trypomastigotes in their salivary glands. The warmest temperature point (A) and mean body temperature (B) were registered with a thermo imager (240 x 180 pixels, Testo 871) over the 132 days of infection and analysed using Testo IRSoft Software 5.0. [generalised estimation equations (GEE); p = 0.04 for warmest temperature point, and p = 0.03 for the mean body temperature]. Data shown as mean **±** s.e. (n = 7-10 for each treatment).

**Fig. 4:**
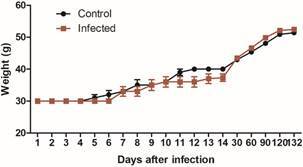
*Trypanosoma rangeli* infection reduces the progressive weight gain of mice [generalised estimation equations (GEE); p = 0.001]. Mice were infected through the bite of *Rhodnius prolixus* nymphs containing metacyclic trypomastigotes in their salivary glands. Data shown as mean **±** s.e. (n = 7-10 for each treatment).

*Effects of infection on body temperature and weight* - Temperature was higher in *T. rangeli*-infected mice in both measurement points (Fig. 3, GEE; Coeff (95% CI) = 0.19 (0.00-0.37), p = 0.04, for warmest temperature point, and Coeff (95% CI) = 0.13 (0.01-0.25), p = 0.03, for the mean body temperature). The body weight of the animals at the end of the experiment did not differ between treatments (Fig 4; GEE; p = 0.33). Nevertheless, the adjusted model showed a significant interaction of time with infection (Table II; p = 0.001). According to the GEE model, this significant interaction indicates that the weight gain of infected mice became slower during the second week post infection and was later compensated in the following two weeks.

**TABLE II:** Variables of the generalised estimating equations (GEEs) with significant effects on the weight of mice infected through the bite of *Rhodnius prolixus* nymphs containing metacyclic trypomastigotes in their salivary glands (n = 7-10 for each treatment)

Predictors	Estimates	CI	p
(Intercept)	33.48	32.48-34.47	< 0.001
Group [Infected]	-1.19	-2.71-0.33	0.125
Days	0.16	0.15-0.17	< 0.001
Group [Infected] × days	0.03	0.01-0.04	0.001

CI: confidence interval.

**Fig. 5:**
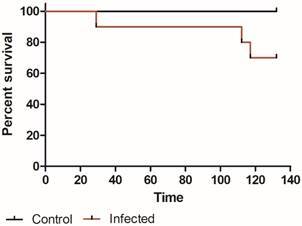
the effect of infection with *Trypanosoma rangeli* on the survival of mice. Mice were infected through the bite of *Rhodnius prolixus* nymphs containing metacyclic trypomastigotes in their salivary glands. (Log-rank test, p = 0.067; n = 10 for each treatment).

*Mice mortality* - Three out of 10 (30%) *T. rangeli*-infected mice died during the 132-day experimental follow-up, while no healthy mice did. Nevertheless, no statistical differences were observed when groups were compared (Fig. 5; Log-rank test, p = 0.067).

## DISCUSSION

The infection of mice through a natural route, specifically the bite of triatomines containing metacyclic forms of *T. rangeli*, produced a persistent infection, with circulating parasites for 90 days. Moreover, the DNA of the parasite was detected in the mesenteric lymph nodes and spleens of all exposed mice, except for mouse #3, where only the lymph nodes tested positive, 132 days after exposure. There are still many controversies about the development of *T. rangeli* in mammals, as its forms of multiplication and the locations where multiplication takes place are not yet known. Herbig-Sandreuter^28^ infected Swiss mice with cultured *T. rangeli* and did not find tissue lesions or intracellular forms of the parasite in several histologically examined tissues. Urdaneta-Morales and Tejero^29^ observed increasing parasitaemia and Scorza et al.^30^ detected a transient presence of amastigote forms in cross-sections of the heart, liver and spleen taken from NMRI lactating mice inoculated with forms of Perro-82 strain culture; these amastigotes disappeared in approximately two weeks. Tanoura et al.^31^ infected ICR mice with trypomastigotes isolated from fresh fibroblast cultures, observing high parasitaemia maintained for about one week, which gradually declined and became undetectable around a month later. No parasites were found in histological analyses of tissues. All cited studies directly inoculated mammals with cultured parasites. In diverse arthropod-pathogen associations, including sandfly-*Leishmania*,^32^ ^,^ ^33^ mosquitoes-arboviruses,^34^ *Anopheles*-*Plasmodium* ^35^ and ticks-*Borrelia*,^36^ saliva can influence transmission to the vertebrate host. Therefore, the transient presence (or direct absence) of parasites observed in the aforementioned studies may be simply due to the inoculation of cultured parasites instead of vector-transmitted forms. It is relevant to consider that parasite form and vector saliva are critical for infection success in other vector-borne systems. In addition, it is worth mentioning that these studies used less sensitive techniques, which may not have detected low parasitic loads. Añez et al.^37^ used the Perro/78 strain of *T. rangeli* to infect mice and opossums through the bite of infected *R. prolixus.* They observed low parasitaemia that became negative 16 and 52 days after infection in mice and opossums, respectively. In our study using natural infection, parasites disappeared from circulating blood after three months, as neither xenodiagnosis nor haemocultures were positive at 120 days post-infection. Nevertheless, the detection of parasite DNA in the spleen and mesenteric lymph nodes by molecular techniques, even when no parasites were present in blood, indicates that the infection remained active.

In nature, *T. rangeli* has been detected by xenodiagnosis, haemoculture, or molecular techniques in diverse mammal species, including humans.^38^ ^,^ ^23^ More recently, Dario et al.^22^ detected *T. rangeli* in 57 samples obtained from haemocultures and blood clots of 1392 mammals captured in Brazil. In the study, the parasite was found infecting 15 mammal species of six orders in five different Brazilian biomes, and for the first time, *T. rangeli* infection was demonstrated in a giant armadillo (*Priodontes maximus*) from the Pantanal biome, which supported the maintenance of the same parasite population with high parasitaemia over a six-month period^22^. Another study found *T. rangeli* in two dogs in Mangaratiba municipality, Rio de Janeiro, Brazil, an area where a case of acute Chagas was identified.^39^

Several studies have demonstrated human infection by *T. rangeli* by direct examination, haemoculture or xenodiagnosis in Venezuela, Guatemala, Panama, Colombia, El Salvador, Costa Rica, Peru and Brazil.^17^ ^,^ ^38^ ^,^ ^40^ Interestingly, with the advancement of molecular techniques, there are several reports of *T. rangeli* identification in samples formerly serologically diagnosed as *T. cruzi*.^41^ ^,^ ^42^ ^,^ ^24^ A case report by Parada et al.^24^ described a 34-year-old female Colombian blood donor, residing in Spain since 2000, who tested reactive for *T. cruzi* in the screening test and in an indirect immunofluorescence assay in 2004, and again tested reactive in the screening test in 2009. This was later confirmed by PCR to be *T. rangeli*. This study is significant as it confirms that the parasite supports long lasting infections producing detectable antibodies and that the cross-reactions with *T. cruzi* can persist for several years. In fact, different studies show that prior immunisation of mice, guinea pigs and dogs with different strains of *T. rangeli* can protect these animals against a virulent strain of *T. cruzi*, resulting in low parasitaemia and increased survival rates.^43^ ^,^ ^44^ ^,^ ^45^

Several non-pathogenic organisms have been used as live vaccine vectors to deliver DNA into cells as efficient delivery tools in gene therapy, including viruses,^46^ bacteria,^47^ and parasites such as *Toxoplasma*,^48^ *Leishmania*,^49^ and *T. cruzi*.^50^ These organisms should be capable of enhancing antigen presentation and eliciting potent immune responses without the risk of development of disease in humans. In this context, *T. rangeli* could be considered a potential candidate as it generates long-lasting antibody levels,^51^ ^,^ ^52^ and apparently does not produce disease in humans. The publication of the genome of *T. rangeli* revealed a reduced diversity of genes associated with mammalian host infection, such as trans-sialidases, MASPs and oxidative stress proteins.^53^ More recently, an *in silico* study of potentially secreted proteins from *T. rangeli*, revealed that the parasite could enhance the production of IL-15, leading to NF-kB complex activation and ultimately an immune response that could affect parasite development in mammals.^54^ In the present study, during the interval in which the infection was monitored, infected mice presented an increase in temperature and slower body weight gain over time when compared to control mice. In addition, three out of ten *T. rangeli*-infected mice died. Although no statistical differences were detected in survival rates, these alterations could indicate that the infection may have negative effects on mammalian hosts. Therefore, these results highlight the need for additional studies to evaluate the humoral and cellular responses generated during *T. rangeli* infection.

Overall, this study provides new insights into the infection dynamics of *T. rangeli* in mammals, specifically mice, through vector-borne transmission. The research confirms that *T. rangeli* establishes a persistent infection in mice, detectable in lymphoid organs long after the parasites disappear from circulating blood. The physiological changes observed in infected mice, such as increased body temperature and slower weight gain, suggest potential subclinical effects of the infection. Therefore, the study highlights the necessity of further investigations into the immune responses elicited by *T. rangeli* infection, considering the potential implications for host health and disease dynamics. Additionally, the findings emphasise the importance of incorporating natural infection routes in experimental designs to better understand parasite behaviour and its interactions with mammalian hosts.
